# Optimisation of medications used in residential aged care facilities: a systematic review and meta-analysis of randomised controlled trials

**DOI:** 10.1186/s12877-020-01634-4

**Published:** 2020-07-08

**Authors:** Hend Almutairi, Andrew Stafford, Christopher Etherton-Beer, Leon Flicker

**Affiliations:** 1 Medical school, University of Western Australia, Perth, Australia; 2 School of Medicine and Pharmacology, Western Australian Centre for Health and Ageing, University of Western Australia , Perth, Australia; 3 Medical School, University of Western Australia, Western Australian Centre for Health and Ageing University of Western Australia, Perth, Australia

**Keywords:** Medication optimisation, Intervention, Elderly, Systematic review, Aged care facility, Clinical outcomes, Meta-analysis

## Abstract

**Background:**

Frail older adults living in residential aged care facilities (RACFs) usually experience comorbidities and are frequently prescribed multiple medications. This increases the potential risk of inappropriate prescribing and its negative consequences. Thus, optimising prescribed medications in RACFs is a challenge for healthcare providers.

**Objective:**

Our aim was to systematically review interventions that increase the appropriateness of medications used in RACFs and the outcomes of these interventions.

**Methods:**

Systematic review and meta-analysis of randomised control trials (RCTs) and cluster randomised control trials (cRCTs) were performed by searching specified databases (MEDLINE, PubMed, Google scholar, PsycINFO) for publications from inception to May 2019 based on defined inclusion criteria. Data were extracted, study quality was assessed and statistically analysed using RevMan v5.3. Medication appropriateness, hospital admissions, mortality, falls, quality of life (QoL), Behavioural and Psychological Symptoms of Dementia (BPSD), adverse drug events (ADEs) and cognitive function could be meta-analysed.

**Results:**

A total of 25 RCTs and cRCTs comprising 19,576 participants met the inclusion criteria. The studies tested various interventions including medication review (*n* = 13), staff education (*n* = 9), multi-disciplinary case conferencing (*n* = 4) and computerised clinical decision support systems (*n* = 2). There was an effect of interventions on medication appropriateness (RR 0.71; 95% confidence interval (CI): 0.60,0.84) (10 studies), and on medication appropriateness scales (standardised mean difference = − 0.67; 95% CI: − 0.97, − 0.36) (2 studies). There were no apparent effects on hospital admission (RR 1.00; 95% CI: 0.93, 1.06), mortality (RR 0.98; 95% CI: 0.86, 1.11), falls (RR 1.06; 95% CI: 0.89,1.26), ADEs (RR 1.04; 95% CI: 0.96,1.13), QoL (standardised mean difference = 0.16; 95% CI:-0.13, 0.45), cognitive function (weighted mean difference = 0.69; 95% CI: − 1.25, 2.64) and BPSD (RR 0.68; 95% CI: 0.44,1.06) (2 studies).

**Conclusion:**

Modest improvements in medication appropriateness were observed in the studies included in this systematic review. However, the effect on clinical measures was limited to drive strong conclusions.

## Background

Inappropriate medication prescription encompasses misprescribing, overprescribing, and underprescribing. Misprescribing involves the use of medication that significantly increases the risk of adverse drug events (ADEs) and involves incorrect dose, frequency, administration and duration. Use of medications that are likely to cause drug-drug interactions or drug-disease interactions is also an aspect of misprescribing. Overprescribing involves the use of medications without clear indications. Underprescribing is the omission of clinically-indicated medication that may have potential benefit for treatment of the disease [1].

Residents of aged care facilities **(**RACFs**)** are often frail and have multiple comorbidities. On average RACF residents take more medications than younger age groups, and more than community-dwelling elderly with similar disease complications [2]. They are frequently prescribed multiple medications that can increase the risk of ADEs, morbidity and mortality [3, 4]. Moreover, the majority of these residents have dementia and the use of psychotropic drugs is typically high. Age-related changes in pharmacodynamics and pharmacokinetics, multiple co-morbidities, and the presence of polypharmacy are the main factors often associated with ageing that makes optimisation of drug therapy a complex task. Furthermore,

Previous studies indicate that about 40% of prescriptions for RACF residents may be suboptimal or inappropriate [5]. Consequently, there is a heightened risk of adverse drug reactions (ADRs), hospitalisations, and medical expense [3, 6]. Therefore, there is an urgent need to improve prescribing and to optimise drug therapy for older people living in care homes [5, 7].

Medication optimisation is a person-centred approach designed to ensure medication safety and improved clinical outcomes via effective use of medicine [8, 9]. A range of interventions for optimisation of prescribed medications in RACFs have been developed to potentially optimise prescribing. These include medication review, education programs, the use of clinical decision support technology, and multidisciplinary case-conferencing. These interventions have been evaluated to determine the effect of optimising prescribing in nursing homes and in older people with dementia, but the results were not pooled statistically [10–12], and the nursing home specific data require updating. The 2011 review concluded that in nursing homes, educational interventions including academic detailing seems to show most promise [12]. The other 2011 review found that education and pharmacist drug review may reduce inappropriate drug use under certain circumstances [11]. The other 2018 review of 18 experimental studies specific to older people living with dementia in any setting concluded that the improvement of medication appropriateness is supported by emerging evidence, and the impact of these interventions on dementia patients’ outcomes required more research [10].

We therefore aimed to systematically review the available interventions conducted by a health professional that aimed to increase the appropriateness of medications used in residential aged care facilities and to evaluate their effects on medication appropriateness and residents’ clinical outcomes.

## Methods

This systematic review was conducted and reported in compliance with the Preferred Reporting Items for Systematic Reviews and Meta-Analyses (PRISMA) guidelines [13]. A PRISMA checklist can be found in Additional file 1. The review was registered with the international prospective register of systematic reviews PROSPERO CRD42020148669.

### Data sources and search strategy

An electronic search of the literature was conducted from inception to May **2019** using the following databases **—** MEDLINE, PubMed, Google scholar, PsycINFO. A combination of the following keywords and MeSH terms were used: “Optimize OR improve OR maximize OR optimization AND medication OR drugs OR medicines AND side effects OR safety OR administration OR review AND nursing homes OR residential OR aged care”. The reference lists of the relevant articles and reviews were hand-searched to further identify any additional studies. The complete search strategy is presented in Additional file 2.

### Study selection

The title and abstract of all retrieved articles were initially reviewed to find those potentially relevant to the study area. The abstract of the selected papers was assessed against five inclusion criteria: (i) randomised controlled trials (RCTs) and cluster randomised controlled trials (cRCTs); (ii) residents’ age 60 and older; settings are residential aged care facility or nursing homes or residential continuing care hospitals; (iii) interventions to increase the appropriateness of medications used in nursing homes (iv) reported in English; (v) published between 1980 and 2019.

### Data extraction and quality assessment

Details of the included articles were independently extracted by two authors (H.A, and L.F). Data included details of the authors, publication year, country, study design, age, setting, sample size, intervention, follow-up, outcomes, and summary of results. Table 1 depicts the study characteristics of the included studies.

**Table 1:** Study characteristics of the included studies

Study ID	Author	Year	Country	Design	Setting	Sample size	Age	Intervention	Follow-up	Outcomes	Summary of results
**1**	Avorn et al. [14]	1992	USA	Cluster RCT	Nursing homes	NHs = 12 NHR = 823	65 and older	- physicians who is there prescribing of psychoactive drugs was above threshold at the baseline evaluation were invited by pharmacists for separate sessions (3 interactive visits). - All physicians of NHR received 6 literature summaries (insomnia, behavioural problems) in 3 mailings. - 4 training sessions were delivered to nurses/ nursing assistants on geriatric pharma psychology, alternatives to psychoactive drugs	5 months	-psychoactive drug use scores -proportion of residents using antipsychotics.	-psychoactive drug use mean in intervention 27% compared to 80% in control group (*P* = 0.02). -antipsychotics ceased in intervention 32% versus 14% in control. - no of days/patient/ month greatly reduced in intervention than control. -no of non-recommended hypnotics ceased and substituted with alternative drugs/discontinued completely were 45% in intervention versus 21% control.
**2**	Rovner et al. [15]	1996	USA	RCT	Nursing homes	1 NH (250 bed community NH) NHR = 89	65 and older	-As ap art of a dementia care program: implementation of new prescribing guidelines based on protocol for psychotropic drug management -Educational rounds weekly for 1 h to discuss patient’s behavioural disorders, and medical status	6 months	- antipsychotic drug - behaviour disorders -restraint use, - and cognitive/ functional status.	-Statistically significant 71% reduction in agitation with intervention versus 49% with control
**3**	Meador et al. [16]	1997	USA	RCT	Nursing homes	NHs = 12 NHR = 1311	65 and older	-geropsychiatrist delivered educational visit to physicians (45-60 min) -NH staff received 5–6 1-h programmes over 1 week delivered by nurse educator. -after 1 month, follow up sessions - when requested, evening meetings for families.	6 months	-Proportion of APs drug use in days/ 100 /days of stay. -severity and presence of behavioural symptoms.	-APs use per 100 days at baseline in intervention gp decreased from 25.3 days to 19.7 per 100. -Aps reduction is 23% in intervention gp to control gp. −33% NHR in intervention gp had their antipsychotics ceased.
**4**	Schmidt et al. 1&2 [17, 18]	1998	Sweden	Cluster RCT	Nursing homes	NH = 33 NHR = 1854	65 and older	-Monthly multidisciplinary meetings led by pharmacist for 12 months	12 months	-Proportion of residents with psychotropics -non recommended hypnotics, antidepressants.	−19% of residents in the intervention gp ceased APs (*p* = 0.007). −37% of residents ceased non- recommended hypnotics in intervention gp (p < 0.001).
	Schmidt et al. [19]	2000	Sweden	Cluster RCT	Nursing homes	NH = 36 NHR = 1549	65 and older	Nursing homes participated in 1995 were followed up.	three-year follow-up	Medication appropriateness	-proportion of residents prescribed non-recommended hypnotics were lower (14.0%) compared to previous study 1995 (19.0%). - in1998 5% of residents were prescribed non recommended hypnotics compared to control gp (10.1%).
	Claesson et al. [20]	1998	Sweden	Cluster-RCT	Nursing homes	NH = 33 NHR = 1854	65 and older	- regular multidisciplinary meetings (physician, pharmacist, NH nurses/assistant) reviewed resident’s drug use on a monthly basis over 12 months. -education for selected pharmacists (5 occasions = 65.5 h), topics were drug use in elderly, geriatrics.	14 months	Medication-related problems	-NH residents were prescribed on average 7.7o (range: 6–11) medications. - laxatives (70%) -psychotropic drugs (77%, range: 50–95%).
**5**	Furniss et al. [21]	2000	England.	Cluster RCT	Nursing homes	NH = 14 NHR = 330 residents: (172 ctrl, 158 Int)	65 and older	**-**Medication review led by pharmacist. - pharmacist review the medications at NH, GPs surgery, or over phone. **-**Pharmacist collected details of current medication, medical history and current problem identified by nursing home staff. - 3 weeks post-medicine review, NH were revisited to identify any problems and to ascertain on whether changes had been implemented.	8 months	-no of prescribed medications -Types of medications, reason for using neuroleptic medications. -hospital admission (in-patient days) -MMSE -GDS -BASDEC -CRBRS -Falls and death	- 239 of recommendations accepted by GP (91.6%). -change of medications =144 -In total MMSE were declined. - Mean CRBRS scores increased in Int compared to ctrl - deaths in ctrl were higher than Int NHs.
**6**	Stein et al. [22]	2001	USA	Cluster RCT	Nursing homes	NH = 20 NHR = 147	65 and older	-Staff training sessions (30 min) -Study physician visited/telephone to all primary care physicians -physicians received messages about NSAIDs risks and benefits , algorithm for stopping NSAIDs, or aternatives such as paracetamol or topical agents and non-pharmacological management for pain.	3 months	NSAIDs and paracetamol Use in the past week	-Mean number of days of NSAIDs use deceased in Int gp from 7.0–1.9 days compared to ctrl gp (7.0–6.2 days), P = 0.0001 - paracetamol use in Int gp increased (3.1 days) compared to ctrl (0.31 days), *P* = 0.0001.
**7**	Roberts et al. [23]	2001	Australia	Cluster RCT	Nursing homes	NH = 52 NHR = 3230	65 and older	-nurse education (6–9 problem-based education sessions) including geriatric medications and common problems in long care such as depression & pain. -supported by bulletins, wall charts and clinical pharmacist visits. - clinical pharmacist average contact 26 h/NH -clinical pharmacist reviewed drug regimen for 500 residents selected by home staff.	12 months	-Mortality rate -hospital admission -Drug use -ADEs -Medication-related problems	-mean no of psycholeptics administered /resident in Int gp decreased (− 0.14,95% CI − 0.28-0.0, *p* = 0.044) - in the intervention group mean number of benzodiazepines Administered/ resident reduced (− 0.06, 95% CI − 0.06 to 0.04, *p* = 0.29).
**8**	Crotty et al. (a) [24]	2004	Australia	Cluster RCT	Aged care facility	NH = 10 NHR = 154	65 and older	−2 multidisciplinary case conferences were conducted 6–12 weeks. -pharmacists, geriatrician, residential care staff, GP, and a representative of the Alzheimer’s Association of South Australia. -medication review prepared by the resident’s GP before case conference.	7 months	-MAI score	-Mean MAI score in Int gp 4.1 (2.1–6.1) versus 0.4 (0.4–1.2) in ctrl gp. - benzodiazepines: mean MAI score in int.gp 0.73 (0.16–1.30) versus − 0.38 (−1.02 to 0.27) in ctrl gp.
**9**	Crotty et al. (b) [25]	2004	Australia	RCT	long-term care facility/hospital discharge	NH = 85 NHR = 110 Discharged from 3 hospitals	65 and older	-pharmacist transition coordinator transfers the medication-related information to the family physician and community pharmacist. -case conference at facility within month of transfer include pharmacist, nurse, family physician, community pharmacist,	8 weeks	-MAI score -Hospital admission -Medication related problems -ADEs, -falls	-No change in MAI score in Int gp 2.5, 95% CI1.4–3.7) -In ctrl gp MAI score had worsened 6.5, 95%CI 3.9–9.1)
**10**	Crotty et al. (c) [26]	2004	Australia	RCT	Residential care facilities	NH = 20 NHR = 715	65 and older	-Educational intervention: two (30 min) outreach visits of pharmacists to doctors. - presenting detailed audit information on psychotropic use, stroke risk reduction, and fall rates. −4 (2 h training sessions) for link nurse in each facility.	7 months	-MAI score -Hospital admissions - MRP	-No significant difference in psychotropic drug use before &after intervention (0.89,95%CI 0.69–1.15). -PRN of antipsychotics drug use increased in Int gp compared to ctrl gp (4.95,95%CI 1.69–14.50). - No significant difference in BZD drug use before & after intervention (0.89,95%CI 0.69–1.15). - No significant difference in falls (1.17, 95%CI 0.86–1.58).
**11**	Fossey et al. [27]	2006	UK	Cluster RCT	Nursing homes	NH = 12 NHR = 349	65 and older	-Training and support to care staff on non-pharmacological interventions, alternatives to neuroleptic use. -Medication review by Led by old age psychiatrist, senior nurse every 3 months -contact between psychiatrist and prescribers to provide and wrote prescribing recommendations	12 months	-Proportion of residents receiving neuroleptics. -CMAI - QoL	- reduction in neuroleptic use/resident (19.1, 95% CI 0.5–37.7%, *P* = 0.045) **--**Neuroleptic use decrease 24% in exp. (47 to 23%) but increased in ctrl 7.6% (49.7 to 42.1%). -No significant changes in CMAI
**12**	Zermansky et al. [28]	2006	UK	RCT	Nursing homes and residential homes	NH = 65 NHR = 661	65 and older	- Pharmacist medication review by using the resident’s medical record. - consultation with the resident’s and carer. -pharmacist forward written recommendations to GP.	6 months	-no. of changes in medication/patient -Hospital admissions -Medication-related problems -Medicine costs -Number of medicines per participant - Mortality - Falls - SMMSE -Barthel index -GP consultations	- Increase in mean number of drug changes/patient ctrl: 2.4 versus 3.1 in Int (P < 0.01) -no of falls reduced significantly - pharmacist recommendations accepted (75.6%), and 76.6% of these recommendations were implemented.
**13**	Gurwitz et al. [29]	2008	USA and Canada	Cluster RCT	Two large long-term care facilities.	Facility = 2 Residents = 1118	65 and older	-Computer program (order entry with clinical decision support system). - more than 600 potentially serious drug-drug interactions alerts were reviewed. -no of ADEs were identified (preventable events including errors and drug-drug interactions were determined). -alerts included in the CDSSs were assessed to determine if any of them could have prevented the prescribing of these drugs.	1 year in one facility and 6 months in the other	-Number of preventable ADEs - ADEs severity - ADEs preventability	-None ADEs = (1.06,95% CI 0.92–1.23) Preventable ADEs = (1.02,95% CI 0.81–1.30)
**14**	Field et al. [30]	2009	Canada	Cluster RCT	long-term care facility	-One long-term care Facility - 22 long-stay units Residents= 833	65 and older	The 22 long-stay units were randomly assigned - for Intervention units’ prescriber: Alerts related to medication prescribing for residents with renal insufficiency were displayed. -Control units: Alerts hidden and tracked - The types alerts were: maximum recommended daily dose/frequency of administration, medication to be avoided, and missing information.	12 months	-Proportion of final drug orders alert that were appropriate	-Appropriate final drug orders proportion were high in Int (1.2, 95% CI 1.0–1.4) for frequency. -for drugs that should be avoided (2.6, 95% CI 1.4–5.0). for missing information (1.8, 95% CI 1.1 to 3.4). -Appropriate final drug orders Significant in Int (1.2 95% CI 1.0–1.4).
**15**	Patterson et al. [31]	2010	Ireland	Cluster RCT	Nursing homes	NH = 11 NHR = 334	65 and older	-intervention homes were visited monthly by trained pharmacists for 1 year. Resident’s information was collected from records, GP and community pharmacist. Interviews were conducted with the residents and next of kin to assess the need for medicines. - applied an algorithm to assess appropriateness of psychoactive medication and worked with GPs to improve the prescribing of these medications.	Monthly for 12 months	-Proportion of residents prescribed inappropriate psychoactive medications. -no of falls	- At 12 months, residents taking inappropriate psychoactive medications in Int gp (19.5%) decreased compared to ctrl gp (50%) intervention homes (0.26, 95% CI 0.14–0.49) -No change the falls rate
**16**	Testad et al. [32]	2010	Norway	Cluster RCT	Nursing homes	NH = 4 NHR = 211	65 and older	-Education and training program (2 days seminar and monthly group guidance for six months).	12 months	-% of residents using antipsychotic drugs - Restraint use	-No statically significant difference in antipsychotic use. - Significant reduction in Aggression in Int gp at 6 & 12 month follow-up. -Significant reduction in proportion of residents restrained at 6 months but not at 12 months.
**17**	Lapane et al. [33]	2011	United States	Cluster RCT	Nursing homes	NH = 25 NHR = 3321	65 and older	- GRAM is automatically generated to assist consultant pharmacists identify residents at risk for delirium/ falls -Detailed instruction of consultant pharmacists providing targeted medication review for all residents at high-risk. - Reports within 24 h of admission and used during monthly review.	12 months	− Mortality − Hospital admission potentially due to ADEs.	-Mortality rate /1000 resident-months, HR: 0.90 (adjusted HR 0.89, 95% CI 0.73–1.08) -Hospital admission/1000 resident-months, HR: 1.13 (adjusted HR 1.11, 95% CI 0.94–1.31).
**18**	Pope et al. [34]	2011	UK, Ireland	RCT	Nurse-managed continuing-care	NHR = 10 nurse-managed continuing-care Residents = 225	65 and older	-medical assessment by a geriatrician, and using Beer’s criteria for multidisciplinary panel medication review. - recommendations forwarded to the GP. - after 6 months, reassessment occurred	6 months	-no of drugs prescribed -mortality -medication cost	−92.7% of patients received medication recommendations and 80.1% accepted. - total number of medications/ patient/d reduced in Int gp (11.64–11.09 compared to ctrl 11.07–11.5).
**19**	Kersten et al. [35]	2013	Norway	RCT	Nursing homes	NH = 22 NHR = 87	65 and older	-A paper-based review with a view to reduce ADS scores were conducted by clinical pharmacist. -clinical pharmacist discuss discontinue or replace an anticholinergic drug with the physician before changes were implemented.	8 weeks	- Cognitive function - anti-cholinergic side-effects	- cognitive function not improved - anti-cholinergic side-effects not improved
**20**	Milos et al. [36]	2013	Switzerland	RCT	Nursing homes or community	NHR = 279	75 years or older	Pharmacists-led medication review that included assessment of relevant parts of (EMRs) and collection of patient’s blood sample data. - clinical pharmacist-initiated medication reviews based on the background information to identify DRPs.	2 months	- no of PIMs. - DRPs	−6% decreased in PIM in Int gp -Total no of DRPs in the intervention group was 431 [mean 2.5 (1.5) / patient (range 0–9) - No significant difference between the no of DRPs in nursing home patients [mean 2.53 (1.33)] and community-dwelling patients [mean 2.55 (1.29)] Significant in changes in the actions taken by the physician were for lowered dosage.
**21**	Frankenthal et al. [37]	2014	Israel	RCT	chronic care geriatric facility	NH = 1 NHR = 359	65 and older	-medication review conducted by pharmacist -to identify PIMs and PPOs medications screened with STOPP/ START criteria then followed up with recommendations to the chief physician. - chief physician decided to accept or not.	12 Months	-medication appropriateness -mortality -hospital admission -QoL -MRP -medication cost	-significant decreased in the average number of drugs prescribed in Int gp (*P* < .001). - significant decreased in the average number of falls in Int gp (*P* = .006). -decrease in the average drug costs in Int gp by US$29. - hospitalization, FIM scores, and QoL were same in both groups.
**22**	García-Gollarte, et al. [38]	2014	Spain	Cluster-RCT	Nursing homes	NH = 36 NHR = 716	65 and older	−30 doctors received educational intervention. - The educational intervention included general drug use in elderly, STOPP START workshop, and adverse drug reactions in older people. -participants also received educational material and references - on-demand support (via phone) for 6 months provided by the educator.	6 Months	- Medication appropriateness (STOPP-START) -Hospital admissions Medication appropriateness (STOPP-START) -Falls	- The mean number of inappropriate drugs was higher in ctrl gp (1.29–1.56) compared to Int gp (0.81–1.13). -no of falls increased in the ctrl gp from 19.3–28% and not significantly change in the intervention group from 25.3–23.9%.
**23**	Pitkala et al. [39]	2014	Finland	Cluster-RCT	Assisted living facilities	Facility = 20 Residents = 227	65 and older	-two 4-h interactive training sessions for nursing staff aimed to enable nurses to recognize potentially harmful medications and corresponding adverse drug events. -the second 4-h sessions: case-study-based. - nurses in this intervention were asked to identify potential MDR and highlight these to the consulting doctor.	12 months	-Medication appropriateness -Hospital admissions -Mortality -QoL -MMSE	-mean number of potentially harmful drugs lowered in int gp (−0.43, 95% CI-0.71 to −0.15) and not changed in ctrl gp (+ 0.11, 95% CI − 0.09 to + 0.31) (*P* = .004). -HR QoL decreased in Int gp (− 0.038, 95% CI − 0.054 to − 0.022) compared to ctrl gp (− 0.072,95% CI − 0.089 to − 0.055) (*P* = .005). -hospital admission decreased significantly in int gp (1.4 days/person/year, 95% CI 1.2 to −1.6) compared to ctrl gp (2.3 days/person/year; 95% CI 2.1to −2.7), RR = 0.60, 95% CI 0.49 to − 0.75, *P* < .001).
**24**	Connolly et al. [40]	2015	New Zealand	Cluster-RCT	RACFs	NH = 36 NHR = 1998	65 and older	- Gerontology nurse specialist delivered staff education and clinical coaching. - benchmarking of resident indicators including restraint use, falls, etc.). - multidisciplinary team meeting (1 h) monthly for the first 3 months.	14 months	- Hospital admissions (ambulatory sensitive hospitalisations, total acute admissions). -Mortality	-no differences between Int and ctrl gp in rates of ambulatory sensitive hospitalisations admission (1.07; 95% CI 0.85–1.36; *P* = 0.59). -no difference in mortality (1.11; 95% CI 0.76–1.61; *P* = 0.62).
**25**	Potter et al. [41]	2016	Australia	RCT	RACFS	Facility = 4 Residents = 95	65 and older	-medication review followed by discontinuing non-beneficial medications conducted by a GP and a geriatrician/clinical Pharmacologist - During deprescribing, the GP reviewed participants weekly.	12 months	-no of falls -mortality -no of fallers -cognitive function -QoL	-mortality 26% in int gp and 40% in ctrl gp (HR 0.60, 95%CI 0.30 to 1.22). -QoL Changes in Int gp (− 1.0 ± 4.3) compared to ctrl gp (− 1.0 ± 4.7). -Falls -Patients with one or more falls in int gp (0.56, 95% CI 0.42–0.69) compared to ctrl gp(0.65, 95% CI 0.50–0.77), (*p* = 0.40)

*Abbreviations*: *RCT* Randomised Controlled Trials, *NHR* Nursing Home Residents, *NHs* Nursing Homes, *CDSSs* Computerised Clinical Decision Support Systems, *CI* Confidence Interval, *GP* General Practitioner, *no* number, *min* minutes, *hr*. hour, *APs* Antipsychotics, *gp* group, *ctrl* control, *Int* intervention, *MMSE* Mini-Mental State Exam, *GDS* Geriatric Depression Scale, *BASDEC* Brief Assessment Schedule Depression Cards, *CRBRS* Crichton-Royal Behaviour Rating Scale, *P*, *p* value, *NSAIDs* Non-Steroidal Anti-Inflammatory Drugs, *ADEs* Adverse Drug Events, *MAI* Medication Appropriateness Index, *MRP* Medication-related Problem, *PRN* pro re nata (when necessary), *BZD* Benzodiazepine, *CMAI* Cohen-Mansfield Agitation Inventory, *QoL* Quality of Life, *exp* experiment group, *%* percentage, *GRAM* Geriatric Risk Assessment Med Guide, *HR* Hazard Ratio, *ADS* Anticholinergic Drug Scale, *EMRs* electronic medical records, *PIMs* Potential Inappropriate medications, *PPOs* potential prescription omissions, *STOPP/START* Screening Tool of Older Person’s potentially inappropriate Prescriptions and Screening Tool to Alert doctors to Right Treatment, *FIM* Functional Independence Measure

### Assessment of risk of Bias

The quality of each article and risk of bias were assessed independently by the two reviewers (H.A, and L.F). For assessing risk of bias we used the Cochrane Collaboration’s tool [42]. The studies assessed based on standard criteria: adequate sequence generation, allocation concealment, blinding of participants and personnel, blinding of outcome assessment, incomplete outcome data, selective reporting, and other bias. Risk of bias tables provided in RevMan v5.3 was used to assess reporting bias. See Risk of Bias in Included Studies Section (Figs. 2 and 3).

### Statistical analysis

Outcome measures such as medication appropriateness, hospitalisation, mortality and other outcomes were assessed for heterogeneity and were pooled for meta-analysis using fixed effects methods if little heterogeneity was found or using random effects methods if heterogeneity was present (*P* value < .05). The software Review Manager (RevMan) version 5.3 (The Cochrane Collaboration, The Nordic Cochrane Centre, Copenhagen, Denmark) was used. The risk ratios (RR) for dichotomous outcomes with 95% confidence intervals (CIs) were calculated. Heterogeneity was measured by *I*^*2*^*.* Continuous outcomes were expressed as mean difference (MD) and standardized mean difference (SMD) between groups with a 95% confidence interval (95% CI). Funnel plots were used to assess possible publication bias (Additional file 3: Figs. S1 to S9). Effect estimates were considered statistically significant of the *p* value was less than 0.05 (2 tailed).

## Results

**Fig. 1:**
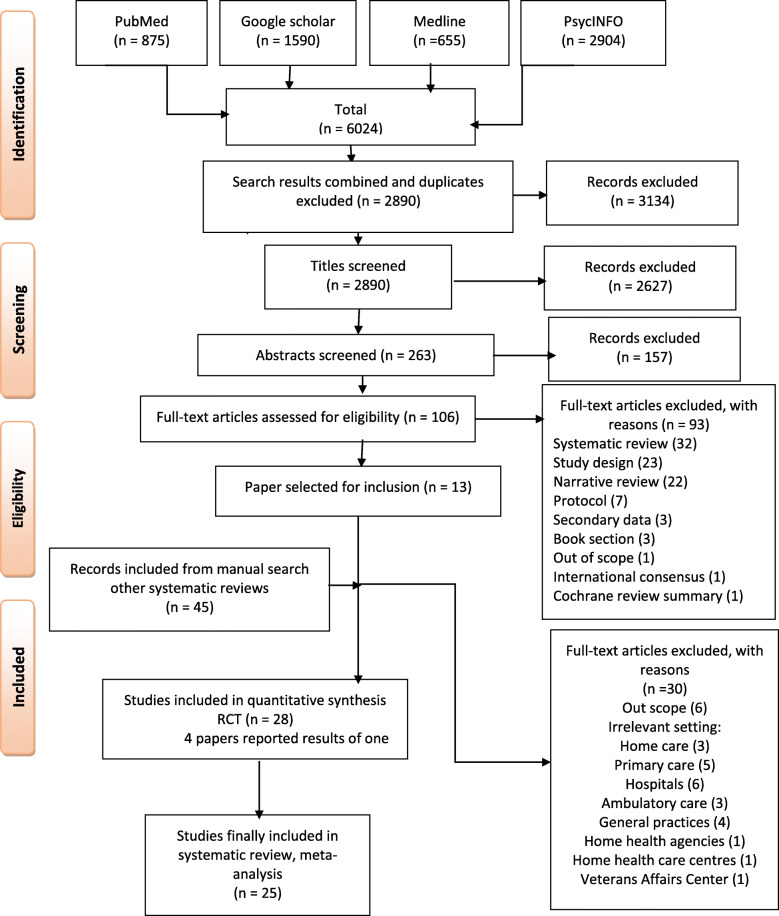
PRISMA flow diagram

The literature search provided a total of 6024 potentially relevant publications. Following independent screening for eligibility, 106 articles were assessed for eligibility of which 25 RCTs and cRCTs were included in this systematic review. The flowchart of the literature search is represented in Fig. 1**.**

### Study design

The design consisted of 15 studies [14, 17, 19–24, 27, 29–33, 38–40] comprising five cRCTs and 10 RCTs [15, 16, 25, 26, 28, 34–37, 41].

### Country and settings

Trials in residential aged care settings or residential continuing care hospitals (long-term care) were conducted in the USA (*n* = 5) [14–16, 22, 33], Australia (n = 5) [23–26, 41], UK (*n* = **3**) [21, 27, 28], Norway (*n* = 2) [32, 35], and one each in Canada [30], Israel [37], Sweden (4 papers reported results of one) [17–20], Finland [39], Spain [38], Switzerland [36], Ireland [31], New Zealand [40] and one combined between USA and Canada [29] and one combined between UK and Ireland [34].

### Participants

Studies included involved older people living in residential aged care facilities aged 60 years and older with a mean age range of 81.2 to 87.2 years.

### Interventions

Various interventions applied by pharmacist, physician or a multidisciplinary team (physicians, pharmacists, and nurses) in the included studies were evaluated. Methods to review residents’ medications were presented in 12 studies [21, 23, 25, 28, 31, 33–38, 41]; nine studies [14–16, 22, 26, 27, 32, 39, 40] investigated the impact of staff education, four studies [17, 20, 24, 25, 40] evaluated the implementation of multi-disciplinary case conferencing, and two studies [29, 30] evaluated computerised clinical decision support systems.

### Outcomes

Most outcome measures in the reviewed studies were reported as (a) medication appropriateness (*n* = 16) [14–17, 19, 23–27, 30, 31, 35–39], hospital admission (*n* = 11) [21, 23, 25, 28, 33, 34, 37–41], mortality (*n* = 9) [21–23, 28, 33, 34, 37, 39, 40], medication-related problems (*n* = 7) [18, 20, 21, 23, 25, 28, 36, 37], falls (n = 7) [26–28, 31, 33, 38, 41], quality of life (*n* = 5) [27, 34, 37, 39, 41], Behavioural and Psychological Symptoms of Dementia BPSD (*n* = 4) [15, 16, 27, 32], ADEs (*n* = 2) [25, 29], and cognitive function (*n* = 2) [15, 35].

#### Medication appropriateness

Medication appropriateness was assessed in 11,470 residents encompassing 16 RCTs [14–17, 19, 23–27, 30, 31, 35–39] by different tools including Medication Appropriateness Index (MAI) [24–26], STOPP-START criteria [37, 38], indicators of appropriate neuroleptic prescribing in nursing homes [27], Beers criteria, Anticholinergic Drug Score (ADS), number of psychotropic medications and non-steroidal anti-inflammatory drugs (NSAIDs( [39].

### Other outcomes

#### Hospital admission

Eleven studies [21, 23, 25, 28, 33, 34, 37–41] specified hospital admission as an outcome measure. Furniss et al. [21] reported in-patient days as hospital admission. Roberts et al. [23] investigated the proportion of hospitalised residents. Crotty et al. [25] reported hospital usage based on unplanned visits to emergency department and hospital readmission. Zermansky et al. [28] reported hospitalisation rate during a 6-month period per resident. Lapane et al. [33] investigated any hospitalisation and potential ADE-related hospitalisation in a randomised cluster trial. Pope et al. [34] reported the number of admissions to acute hospital. Frankenthal et al. [37] reported hospital admissions. Garcia-Gollarte et al. [38] reported the total number of days spent in hospital. Pitkala et al. [39] reported hospital days/resident/year. Connolly et al. [40] reported all acute admissions and ambulatory sensitive hospitalisations. Potter et al. [41] reported hospital admission as the proportion of residents experiencing an unplanned hospital admission.

#### Mortality

Nine studies [21–23, 28, 33, 34, 37, 39, 40] included mortality as an outcome measure. Furniss et al. [21] reported mortality as a number of deaths over 8-months,by Zermansky et al. [28] over 6-months. The number of deaths was reported by Stein et al. [22] over a 3-month evaluation period, by Pope et al. [34] over a 6-month period, and by Frankenthal et al. [37] over 1 year. Roberts et al. [23] reported residents’ cumulative survival and death proportion for 1 year. Lapane et al. [33] calculated the average percentage of mortality per 1000 person-months. Pitkala et al. [39] used a Cox proportional hazard model to calculate hazard ratios. Connolly et al. [40] reported death risk ratio over 14 months.

#### Falls

Seven studies [26–28, 31, 33, 38, 41] included falls as an outcome measure. Crotty et al. [26] calculated the percentage of residents who fell in 3 months prior. Fossey et al. [27] reported the proportion of residents who had at least one fall over a 12-month period. Zermansky et al. [28] reported number of falls over 6 months. Patterson et al. [31] calculated falls rate per 100 resident-months. Lapane et al. [33] reported the number of people falling over 12 months. Garcia-Gollarte et al. [38] reported the number of falls and fallers post-intervention. Potter et al. [41] reported the proportion of patients with one or more falls.

#### Medication - related problems

Seven studies [18, 20, 21, 23, 25, 28, 36, 37] included medication - related problems as an outcome measure. Claesson and Schmidt et al. [18, 20] reported the type and frequency of drug-related problems discussed by clinical teams and their recommendations. Furniss et al. [21] reported the total number of recommendations made by the pharmacist, and the accepted recommendations by the general physician (GP) and the actual changes in medications. Roberts et al. [23] calculated the number of drug changes. Crotty et al. [25] categorised medication-related problems to different categories such as high dose, administration time and no indication. Zermansky et al. [28] measured the recommendations made by the pharmacist against the number of accepted/rejected recommendations of the doctor. Milos et al. [36] measured the percentage of medications changed. Frankenthal et al. [37] measured the number of recommendations accepted by the GP according to the STOPP-START criteria. There was no extractable data for this outcome and therefore meta-analysis was not performed.

#### Quality of Life (QoL)

Five studies [27, 34, 37, 39, 41] reported patient Quality of Life (QoL). Fossey et al. [27] reported rating for wellbeing in residents. Pope et al. [34] measured QoL by asking patients with Abbreviated Mental Test Score (AMTS ≥8) or staff who were familiar with the patient about whether the intervention had been of benefit. Frankenthal et al. [37] used the Medical Outcomes Study 12-item Short-form Health survey (SF-12). Pitkala et al. [39] used the 15-dimensional instrument of health-related QoL (15D). Potter et al. [41] used self-reported QoL assessed with Quality of Life in Alzheimer’s Dementia (QOLAD).

#### Behavioural and Psychological Symptoms of Dementia (BPSD)

Four studies [15, 16, 27, 32] assessed Behavioural and Psychological Symptoms of Dementia (BPSD). Rovner et al. [15] reported behaviour disorder. Fossey et al. [27] reported aggression events in past 12 months and Testad et al. [32] used the Cohen-Mansfield agitation inventory tool (CMAI) to measure agitated behaviour of residents. Meador et al. [16] used Nursing Home Behaviour Problem Scale (NHBPS).

#### ADEs

Two studies [25, 29] reported ADEs. One study defined ADE as an injury resulting from the use of a drug [29]. Crotty et al. [25] investigated number of ADEs during the 8-week follow-up period.

#### Cognitive function

Two studies [15, 35] included cognitive function as an outcome measure. Rovner et al. [15] used the Norwegian version of the global cognitive test Mini-Mental Sate Examination (MMSE) to assess cognition. Kersten et al. [35] used the Consortium to Establish a Registry for Alzheimer’s Disease (CERAD)‘s 10-word list test for delayed recall and recognition and MMSE.

### Risk of bias in included studies

**Fig. 2:**
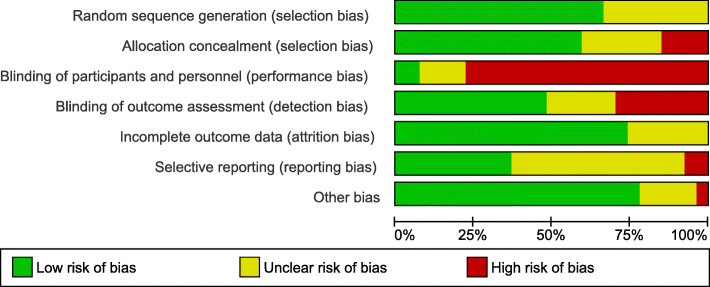
Risk of bias graph

**Fig. 3:**
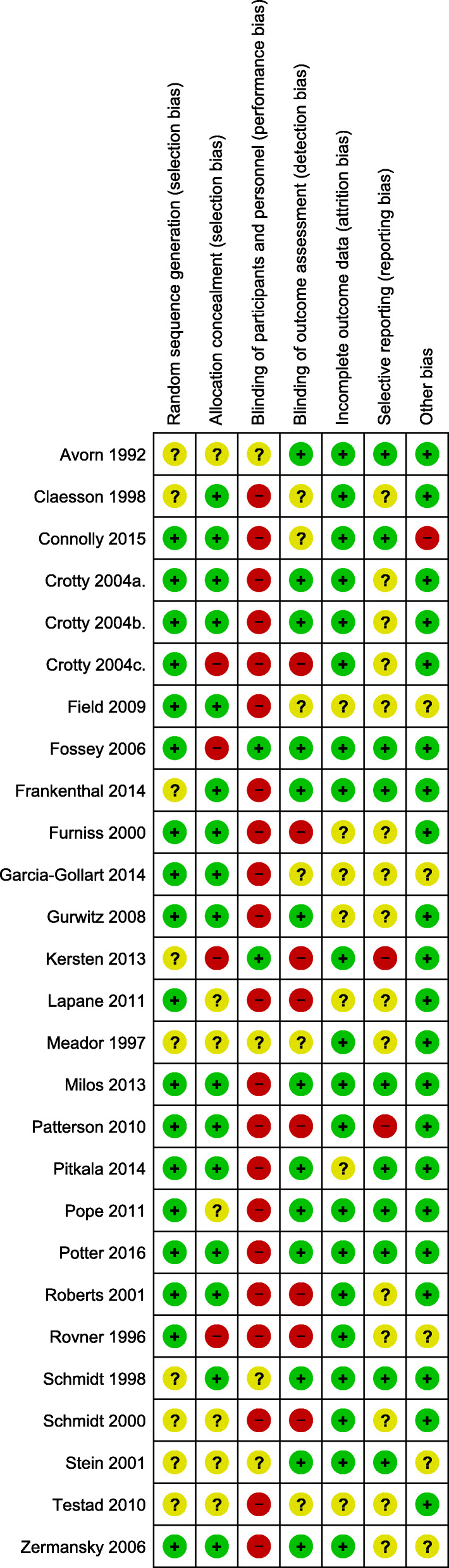
Risk of bias summary

Assessment of the risk of bias is summarised in (Figs. 2 and 3). Except for nine studies with unclear risk [14, 16, 17, 19, 20, 22, 32, 35, 37], the remaining 16 studies had low risk of selection bias. Performance bias was high in 21 (77.8%) studies [15, 19–21, 23–26, 28–34, 36–41]; detection bias was high in eight (29.6%) studies [15, 19, 21, 23, 26, 31, 33, 35], and allocation concealment was found in four (14.8%) studies [15, 26, 27, 35]. In most of the studies, blinding of participants and staff was not possible due to the nature of the intervention.

### Effectiveness of the interventions

#### Medication appropriateness

**Fig. 4:**
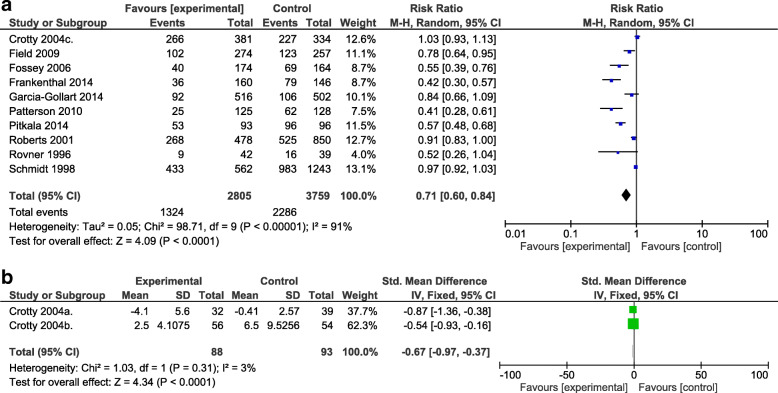
**a**: A meta-analysis of the effect of interventions on medication appropriateness. **b**: Standardised mean difference in the change of MAI score comparing experimental (intervention) group and control group

**Fig. 5:**
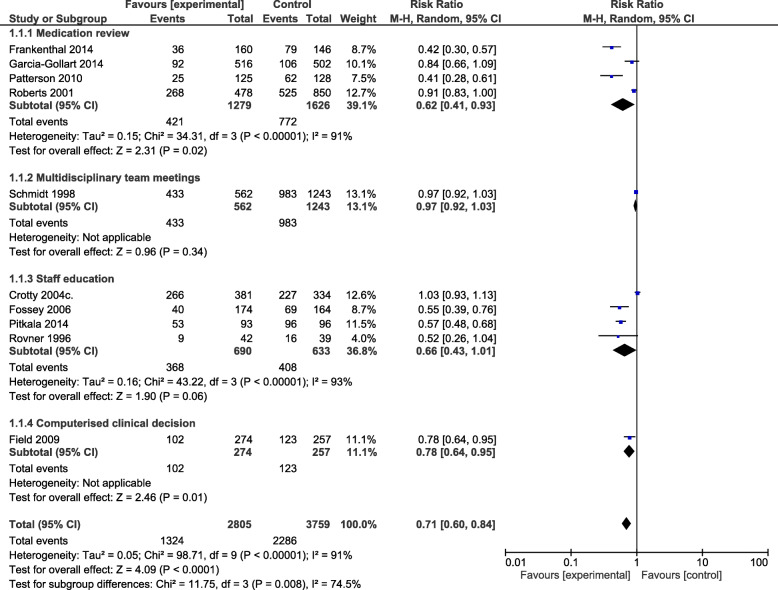
Subgroup analysis of the effect of interventions medication review, multi-disciplinary team meetings, staff education and computerised clinical decision

Meta-analysis of medication appropriateness (Fig. 4) including 6754 residents [15, 17, 23–27, 30, 31, 37–39] showed a significant improvement on medication appropriateness (RR 0.71; 95% confidence interval (CI): 0.60, 0.84, despite high heterogeneity (*P* <  0.00001; *I*^2^ = **91**%). This outcome was assessed for the intervention subtypes of staff education (RR 0.66, 95% CI:0.43, 1.01), implementation of multi-disciplinary case conferencing (RR 0.97,95% CI:0.92, 1.03) computerised clinical decision support systems (RR 0.78, 95% CI:0.64, 0.95) and medication review (RR 0.62 95% CI:0.41, 0.93) (See Fig. 5).

The standardised mean difference of medication appropriateness scales for the remaining two Crotty et al. studies [24, 25] was calculated separately (standardised mean difference = − 0.67; 95% CI: − 0.97, − 0.36) with a heterogeneity of *I*^2^ = 3%.

### Other outcomes

#### Hospital admission

**Fig. 6:**
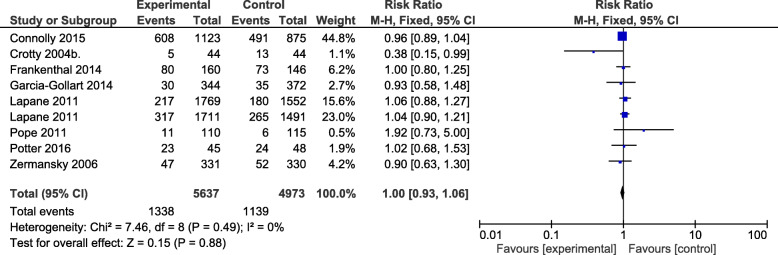
A meta-analysis of the effect of interventions on hospital admission

Meta-analysis of hospital admission **(**Fig. 6**)** as an outcome measure investigated in 11,272 residents resulted in the analysis of eight studies [25, 28, 33, 34, 37, 38, 40, 41] (10,610 residents), which showed that interventions have no effect on hospital admission RR = 1.00, 95% CI: 0.93,1.06) with a heterogeneity of *I*^2^ = 0%.

#### Mortality

**Fig. 7:**
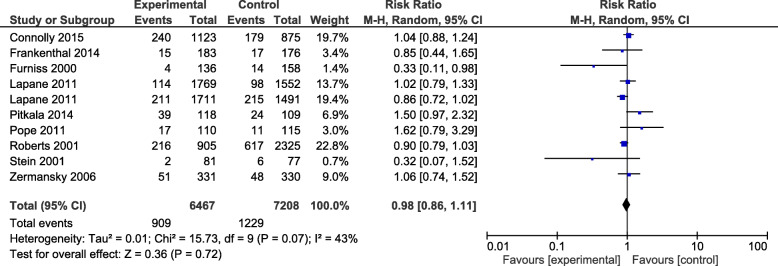
A meta-analysis of the effect of interventions on mortality

Meta-analysis of mortality (Fig. 7) as an outcome measure investigated in 13,675 residents [21–23, 28, 33, 34, 37, 39, 40] showed no significant difference between the intervention group and control group (RR 0.98, 95% CI: 0.86,1.11, *P* = 0.07) with a heterogeneity of *I*^2^ = 43%.

#### Falls

**Fig. 8:**
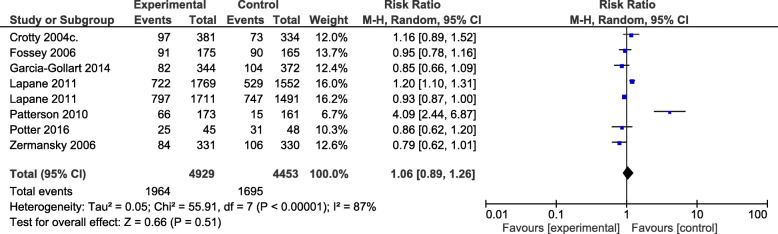
A meta-analysis of the effect of interventions on falls

Meta-analysis of falls (Fig. 8) as an outcome measure investigated in 9382 residents [26–28, 31, 33, 38, 41] showed that interventions had no effect on falls (RR = 1.06; 95%CI: 0.89,1.26) with a heterogeneity of *I*^2^ = 87%.

#### Medication- related problems

Claesson and Schmidt et al. [18, 20] found 819 medication -related problems in 395 participants resulting in an action taken in 90% (737) with withdrawal of a drug in 368 (45%) and change of medications in 162 (20%). Furniss et al. [21] reported that 239 (92%) of 261 recommendations were accepted by the GP resulting in change in therapy in 144 patients. The most common reasons for recommendations (33%) were the medication indication was no longer present. Roberts et al. [23] found that medication reviews resulted in changes to medications in 54 (39%) of residents. Crotty et al. [25] reported that the most common medication-related problem identified in control and intervention groups was that the residents were allocated to a new family physician when transfer to long-term care facility (*n* = 35, 62.5% intervention; *n* = 41, 75.9% control). Zermansky et al. [28] found a significant difference in the mean number of drug changes per resident (mean 3.1, SD = 2.7 for intervention and mean 2.4, SD = 2.6 for control) (*P* <  0.0001). Milos et al. [36] found similar number of drug-related problems between community-dwelling patients (mean 2.55, SD = 1.29) and nursing home residents (mean 2.53, SD = 1.33) (*p* = 0.767). 56% of drug-related problems resulted in an action taken and change of medications (mean 1.44, SD = 1.33) with no difference between the community dwelling and the nursing home patients (*p* = 0.946). Frankenthal et al. [37] made 327 recommendations - 245 in 129 residents based on STOPP and 82 in 65 residents based on START. The physician accepted 82.4% of STOPP recommendations and 92.6% of START recommendations.

#### Quality of Life (QoL)

**Fig. 9:**
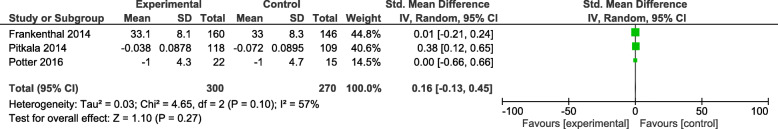
A meta-analysis of the effect of interventions on quality of life (QoL)

Meta-analysis of quality of life (Fig. 9) outcomes in 570 residents, of a total of 1141 residents that included QoL as an outcome measure, found that the interventions had no effect on residents’ QoL (standardised mean difference = 0.16 95% CI:-0.13, 0.45) with a heterogeneity of *I*^2 =^ 57% between trials [37, 39, 41]. Pitkala et al. [39] reported that health-related QoL in the intervention group (− 0.038, 95% CI: − 0.054, − 0.022) declined more slowly than in control group during 12-month follow-up (− 0.072, 95% CI: − 0.089, − 0.055). Frankenthal et al. [37] found no significant difference between groups in the physical average score (intervention mean 33.1 ± 8.1, control mean 33 ± 8.3, *p* = 0.09) and mental components (intervention mean 37.7 ± 1.7, control mean 39.6 ± 11.3 *p* = 0.70) of the SF-12 questionnaire.

#### BPSD

**Fig. 10:**
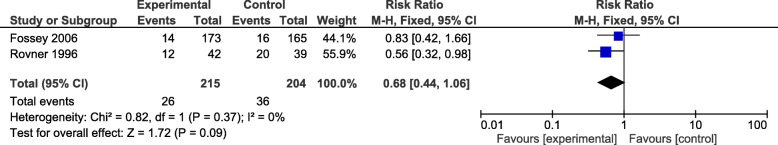
A meta-analysis of the effect of interventions on Behavioural and Psychological Symptoms of Dementia (BPSD)

Of 1941 residents examined, pooled analysis of BPSD of two studies [15, 27] comprising 419 residents showed no significant change after the intervention (RR 0.68, 95% CI: 0.44,1.06; *I*^2 =^0%) (Fig. 10).

#### ADEs

**Fig. 11:**
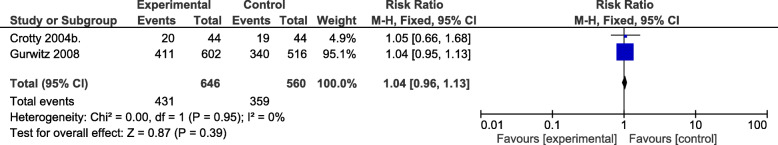
A meta-analysis of the effect of interventions on adverse drug events (ADEs)

Of the 1206 residents [25, 29] examined for ADEs after the intervention. Neither of the two RCTs reported any statistically significant differences in ADEs between the intervention and control groups. The RR for all ADEs was 1.04 (95% CI: 0.96,1.13; *I*^2 =^0%) (Fig. 11).

#### Cognitive function

**Fig. 12:**
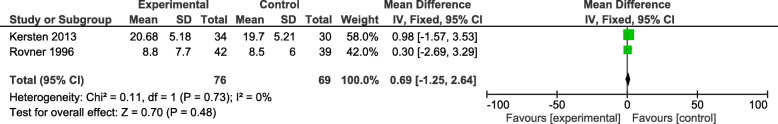
A meta-analysis of the effect of interventions on cognitive function

Meta-analysis of 145 residents [15, 35] indicated that the interventions had no effect on cognitive function (weighted mean difference = 0.69, 95%CI: − 1.25, 2.64) (Fig. 12). No heterogeneity was detected between trials (*I*^2 =^0).

## Discussion

This systematic review examined how a wide variety of interventions optimise medications prescribed in nursing homes, when applied either individually or through multi-faceted approaches. Our meta-analysis of available data showed that the interventions implemented in the included studies can improve medication appropriateness in older residents, although heterogeneity was high among included studies. Whilst these results were promising, the impact on the residents’ clinical outcomes was undetectable. There was limited evidence for effectiveness of interventions in reducing hospitalisation, all-cause mortality, falls, ADEs, cognitive function or BPSD.

There are several published systematic reviews on clinical outcomes of different interventions conducted in aged care homes. In a review by Forsetlund and colleagues [11], the authors found that both educational outreach/educational interventions and medication review by pharmacists under certain situations could reduce inappropriate drug use in nursing homes. However, they reported that the evidence was of poor quality and too low to assess the effect of the interventions on health outcomes. A review by Loganathan et al. [12] grouped the interventions into four groups (staff education including academic detailing, multi-disciplinary team meetings, medication review, and computerised clinical decision support system). No one interventional strategy was found to be effective. However, the most promising intervention seems to be education including academic detailing. That review reported that multifaceted interventions are likely to be required to improve prescribing in care homes [12]. A narrative review by Shafiee et al. [10], which included 18 studies, seven of them RCTs, found that the interventions may improve medication appropriateness in people with dementia in any settings, but the evidence for the effect of the interventions on health outcomes remained uncertain.

Our findings on clinical outcomes are in line with that of previous reviews [43, 44], which found no evidence for the interventions impacting resident’s clinical outcomes such as ADEs, mortality, QoL and hospital admission. Since the elderly often exhibit non-specific clinical symptoms such as depression, constipation, falls and confusion, it is difficult to detect ADEs as opposed to the general condition of the residents. Another possible reason for the lack of significant effect of interventions on falls and ADEs may be the potential for underreporting of incidents that were obtained from nursing records. The lack of effect of interventions on QoL in the treatment group compared to the control may be attributed to the wide variation in the length of the follow up period (3–12 months) [43].

Interventions that focus on individual team members may had limited effectiveness in busy clinical environment. For example, the educational intervention delivered by a pharmacist [26] failed to have any significant effect on major outcomes. The investigators demonstrated that the lack of effect was attributed to staff attrition, short study duration, and not all the physicians in the recruited homes participated in the study.

Very few of the interventions were based on strong theoretical foundations. An exception was the educational study by Pitkälä et al. [39], who suggested that the use of constructive learning theory to recognise potentially harmful medications was more likely to change practice in healthcare than using lectures alone. Deficits in the education of health careworkers were thought to be important. Forsetlund et al. [11] suggested that health care providers receive inadequate training in geriatric medications in their education. Therefore, any intervention for minimising medications usually requires some form of education.

Although our primary focus was on clinical outcomes, some interventions demonstrated a decrease in medication-related costs. Frunsis et al. [21] reported a reduction in the cost of medicine per resident over 4 months period by 27.47 GB Pounds in the intervention group. Roberts et al. [23] reported savings in drug cost (64 AUD/ resident/year in intervention group) in the clinical pharmacy program. Frankenthal et al. [37] found a significant reduction in the average monthly costs of medications in the intervention group ILS 279 ± 171.9 compared to baseline ILS 382.7 ± 279 (*P* < 0.001) at 12 months follow-up period. Pope et al. [34] reported a net reduction in medication cost in intervention group over a 6-month period. While, Crotty et al. [24] reported similar Pharmaceutical Benefit Scheme (PBS) monthly drug costs of regular medications between groups (mean AUD 359 in intervention versus AUD 303 in control (*P* = 0.837). These interventions require resources and therefore, evaluating these interventions economically and their cost-effectiveness should be considered in future research.

### Strengths and limitations

This systematic review was based on a comprehensive search of the literature that was limited to 25 studies with robust design (RCT, cRCT) and compared to previous reviews on related topics [12, 44], our sample size may be regarded as sufficiently powered.

Another strength of this review was a focus only on residents in care homes. The nursing home population is at heightened risk of receiving multiple drugs because of their comorbidities. Therefore, evaluating specific available interventions optimising medications in this setting is required. We were able to complete meta-analyses to pooled the overall effects.

This study is not without limitations. We included only English language publications, which may lead to potential omission of other interventions. Although MEDLINE, PubMed, Google scholar, and PsycINFO databases were searched for relevant articles, some studies indexed in other databases may have been missed. We identified several additional articles manually which may indicate poor indexing of older studies and a lack of consistent terminology.

Due to the nature of the interventions, performance and detection biases may have resulted from the difficulty in maintaining blinding. Meta-analysis of some studies was difficult due to the variations in the measurement of specific outcomes. Certain outcomes, such as cognitive function, were examined in a limited number of articles, reducing the power of the analysis. Further, certain studies were small or had short study periods, which may potentially limit the effect of an intervention on the outcomes. We attempted to evaluate medication-related problems such as drug interactions, number of pharmacist recommendations etc. but these outcomes were not consistently reported in the studies and this our ability to draw any robust conclusions was limited.

Heterogeneity was notable among some studies included in the meta-analysis. The factors that caused this heterogeneity were difficult to discern. Due to differences in training, the characteristics of the nursing home residents, healthcare culture, the number of physicians’ visits and their usual practices the ability to generalise findings from one country to another is difficult.

### Implications for research and practice

In view of the considerable investment in strategies aimed at improving medication appropriateness in RACFs worldwide, our findings question the value of such interventions based on the apparent lack of outcomes that may be meaningful to RACF residents.

Large, high quality RCT studies are required to identify effective interventions to optimise medications used in RACFs. Regarding physicians or staff acceptance of the intervention, only limited information was provided in the studies. Further qualitative study utilising semi-structured interviews may provide useful information to obtain the opinion of healthcare professionals with regard to process outcomes, such as whether the intervention was perceived to be successful or not, and to identify the potential means to overcome the barriers to changing professional behaviour by this method. More intensive interventions on medical and care staff with more stringent monitoring may be required.

## Conclusion

This systematic review found that multifaceted interventions including medication review, staff education/training, multi-disciplinary case-conferencing and clinical decision support technology could improve the appropriateness of medications at RACFs. However, evidence for the effect of these interventions on residents’ clinical outcomes was scarce and no conclusion could be drawn. More robust clinical studies are required to ascertain the health outcomes benefits.

## Supplementary information

**Additional file 1.** PRISMA checklist.

**Additional file 2.** Search Strategy.

**Additional file 3 **Funnel plots (pdf). **Figure S1.** Medication appropriateness funnel plot (10studies). **Figure S2.** Medication appropriateness funnel plot (2 studies). **Figure S3.** Hospital admission funnel plot. **Figure S4.** Mortality funnel plot. **Figure S5.** Falls funnel plot. **Figure S6.** Quality of life (QoL) funnel plot. **Figure S7.** Behavioural and Psychological Symptoms of Dementia (BPSD) funnel plot. **Figure S8.** Adverse drug events (ADEs) funnel plot. **Figure S9.** Cognitive function funnel plot.

## Data Availability

The datasets supporting the conclusions of this article are included within the article and its additional files.

## Abbreviations

RACFs: Residential aged care facilities
RCTs: Randomised control trials
cRCTs: Cluster randomised control trials
QoL: Quality of life
BPSD: Behavioural and Psychological Symptoms of Dementia
ADEs: Adverse drug events
CI: Confidence interval
RR: Risk ratio
MD: Mean difference
SMD: Standardized mean difference
ADRs: Adverse drug reactions
PRISMA: Preferred reporting items for systematic reviews and meta-analyses
MAI: Medication appropriateness index
ADS: Anticholinergic drug score
NSAIDs: Non-steroidal anti-inflammatory drugs
AMTS: Abbreviated mental test score
15 D: 15-dimensional instrument of health-related QoL
QOLAD: Quality of Life in Alzheimer’s Dementia
CMAI: Cohen-Mansfield agitation inventory tool
NHBPS: Nursing Home Behaviour Problem Scale
MMSE: Mini-Mental Sate Examination
CERAD: Consortium to Establish a Registry for Alzheimer’s Disease
PIP: Potentially inappropriate prescriptions
PBS: Pharmaceutical Benefit Scheme
