# Supplementary material for: Optimisation of medications used in residential aged care facilities: a systematic review and meta-analysis of randomised controlled trials
Source: BMC Geriatr. 2020 Jul 8;20:236. doi: 10.1186/s12877-020-01634-4 (PMC7346508; doi:10.1186/s12877-020-01634-4)
Supplement: Supplementary file 2 — Additional file 2. Search Strategy. [file 12877_2020_1634_MOESM2_ESM.docx]

**Additional file 2. Search Strategy**

An electronic search of the literature was conducted from inception to May 2019 using the following databases — MEDLINE, PubMed, Google scholar, PsycINFO. A combination of the following keywords and MeSH terms were used:

optimize or improve or maximize or optimization AND

medications or drugs or medicines AND

side effects or safety or administration or review AND

homes nursing or residential or aged care

Filters:

Systematic Reviews or Randomized Controlled Trial or Observational Study

1980- 2019

Results:

**Pubmed (875 articles)**

Search ((((optimize or improve or maximize or optimization)) AND (medications or drugs or medicines)) AND (side effects or safety or administration or review)) AND (homes nursing or residential or aged care) Sort by: Publication Date Filters: Systematic Reviews; Randomized Controlled Trial; Observational Study; Publication date from 1980/01/01 to 2019/12/31

**Google scholar (1,590 articles)**

optimize or improve or maximize or optimization medications or drugs or medicines side effects or safety or administration or review homes nursing or residential or aged care Systematic Reviews; Randomized Controlled Trial; Observational Study

Filter 1980-2019

**PsyclNFO (2904 articles)**

Search history sorted by Search number ascending

- # Searches
- 1. (optimize or improve or maximize or optimization medications or drug or medicines side effect safely or administration or ‘reviews homes nursing or resent of aged care). mp.[mp=title, abstract, Heading word, table of content, key concepts, original, title, tests & measures)
- 2. limit 1 (human and English language and (“0300 clinical trial” or "0830 systematic review”) and"380 aged <age 6S yrs and older" and older>”0110 peer-reviewed journal” and yr="1980-2019")

**OR**

**Save**

**Remove**

Combine with:

**AND**

**Medline (655 articles)**

1. (optimize or improve or maximize or optimization medications or drugs or medicines side effects or safety or administration or review homes nursing or residential or aged care).mp. [mp=title, abstract, original title, name of substance word, subject heading word, floating sub-heading word, keyword heading word, organism supplementary concept word, protocol supplementary concept word, rare disease supplementary concept word, unique identifier, synonyms]
2. limit 1 to (english language and full text and humans and yr="1980 - 2019" and "all aged (65 and over)" and (observational study or randomized controlled trial or "systematic review"))
3. limit 2 to "reviews (maximizes sensitivity)"
4. limit 3 to “reviews (maximizes specificity)"
