# Supplementary figures and images for: Optimisation of medications used in residential aged care facilities: a systematic review and meta-analysis of randomised controlled trials
Source: BMC Geriatr. 2020 Jul 8;20:236. doi: 10.1186/s12877-020-01634-4 (PMC7346508; doi:10.1186/s12877-020-01634-4)

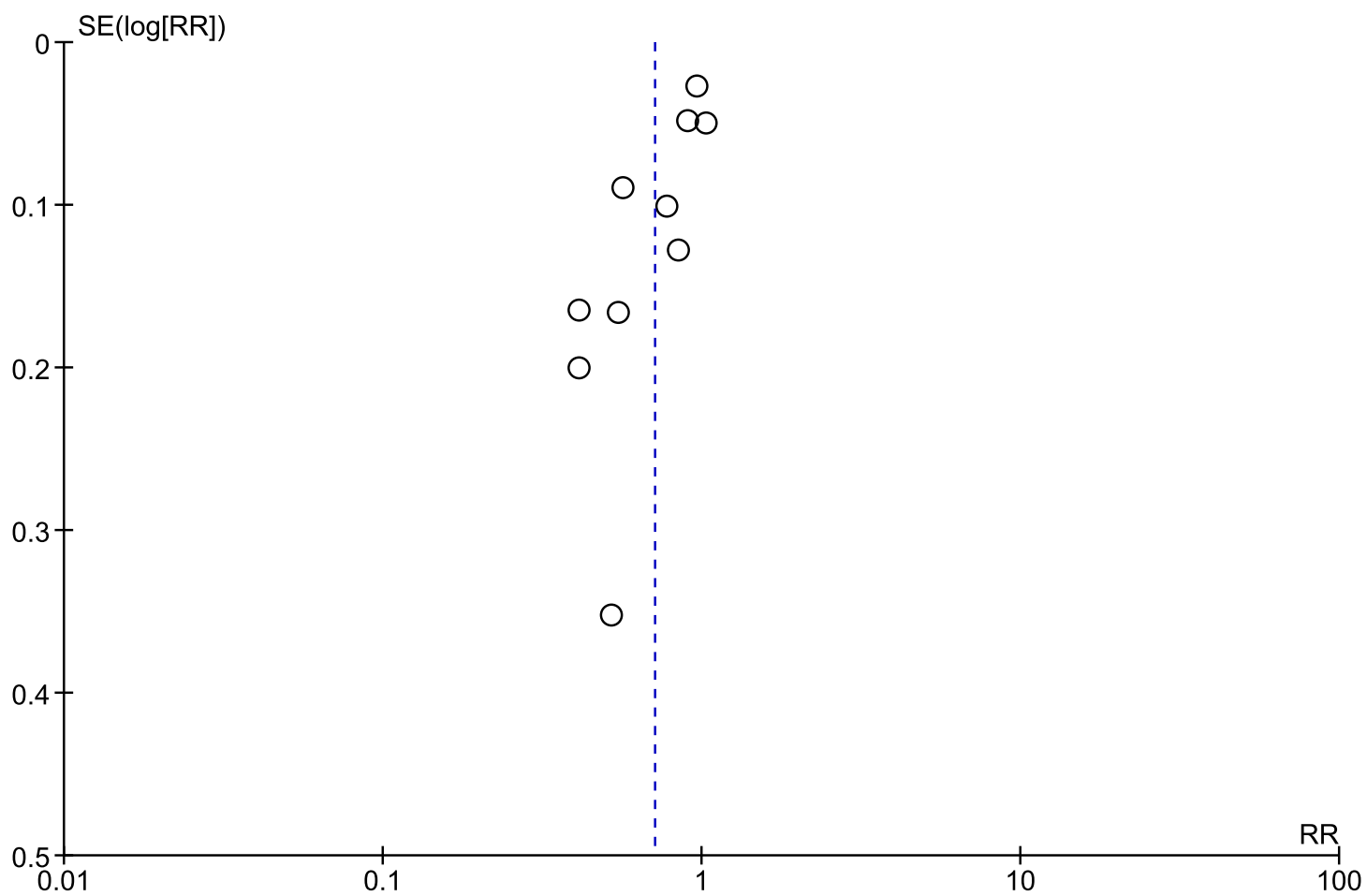

Figure S1. Medication appropriateness funnel plot (10studies).

Supplement: Supplementary file 3 — Additional file 3 Funnel plots (pdf). Figure S1. Medication appropriateness funnel plot (10studies). Figure S2. Medication appropriateness funnel plot (2 studies). Figure S3. Hospital admission funnel plot. Figure S4. Mortality funnel plot. Figure S5. Falls funnel plot. Figure S6. Quality of life (QoL) funnel plot. Figure S7. Behavioural and Psychological Symptoms of Dementia (BPSD) funnel plot. Figure S8. Adverse drug events (ADEs) funnel plot. Figure S9. Cognitive function funnel plot. [file 12877_2020_1634_MOESM3_ESM.zip › Figure S1. Medication appropriateness AR3.pdf]

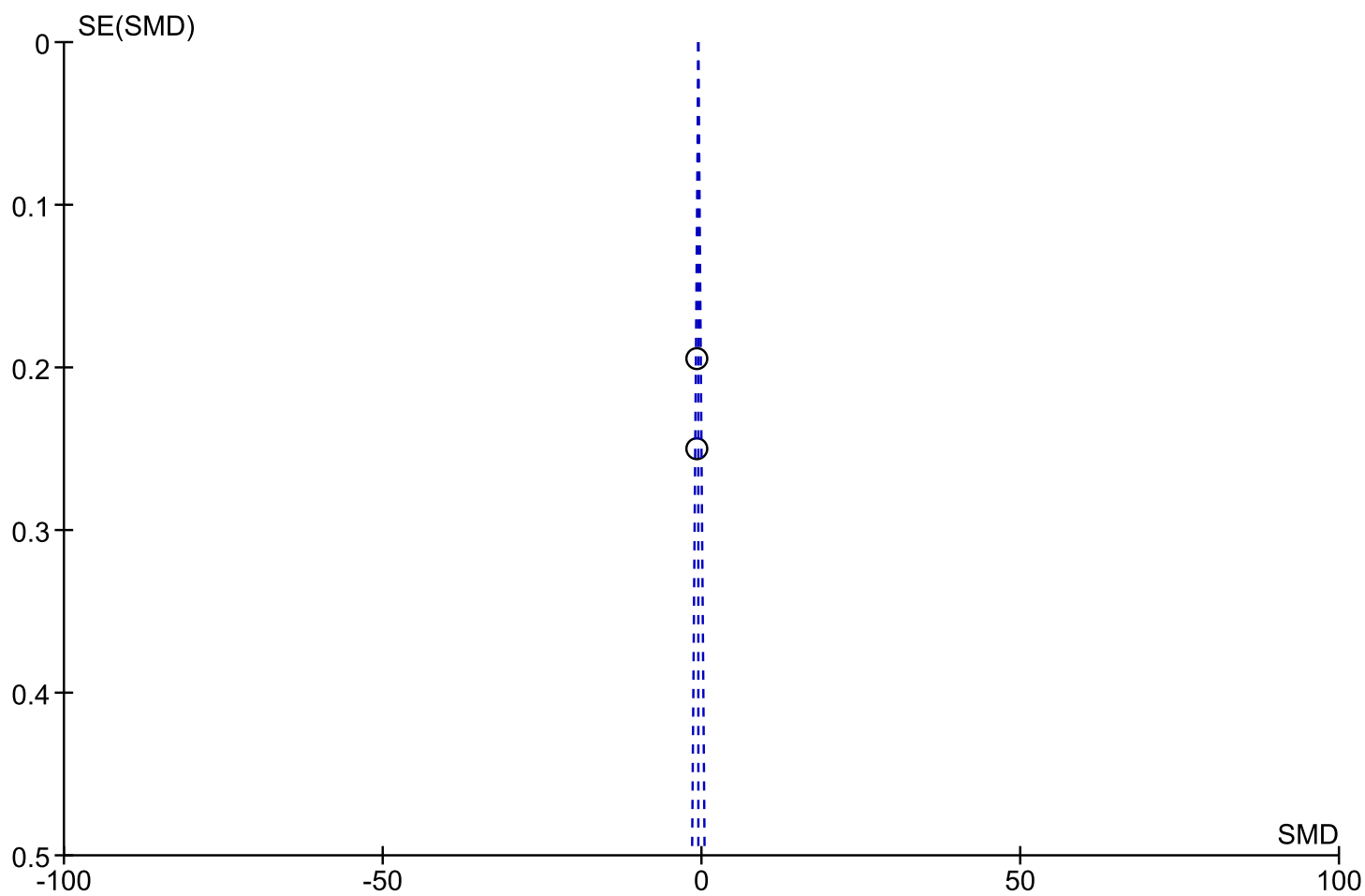

Figure S2. Medication appropriateness funnel plot (2 studies).

Supplement: Supplementary file 3 — Additional file 3 Funnel plots (pdf). Figure S1. Medication appropriateness funnel plot (10studies). Figure S2. Medication appropriateness funnel plot (2 studies). Figure S3. Hospital admission funnel plot. Figure S4. Mortality funnel plot. Figure S5. Falls funnel plot. Figure S6. Quality of life (QoL) funnel plot. Figure S7. Behavioural and Psychological Symptoms of Dementia (BPSD) funnel plot. Figure S8. Adverse drug events (ADEs) funnel plot. Figure S9. Cognitive function funnel plot. [file 12877_2020_1634_MOESM3_ESM.zip › Figure S2. Medication appropriateness BR3.pdf]

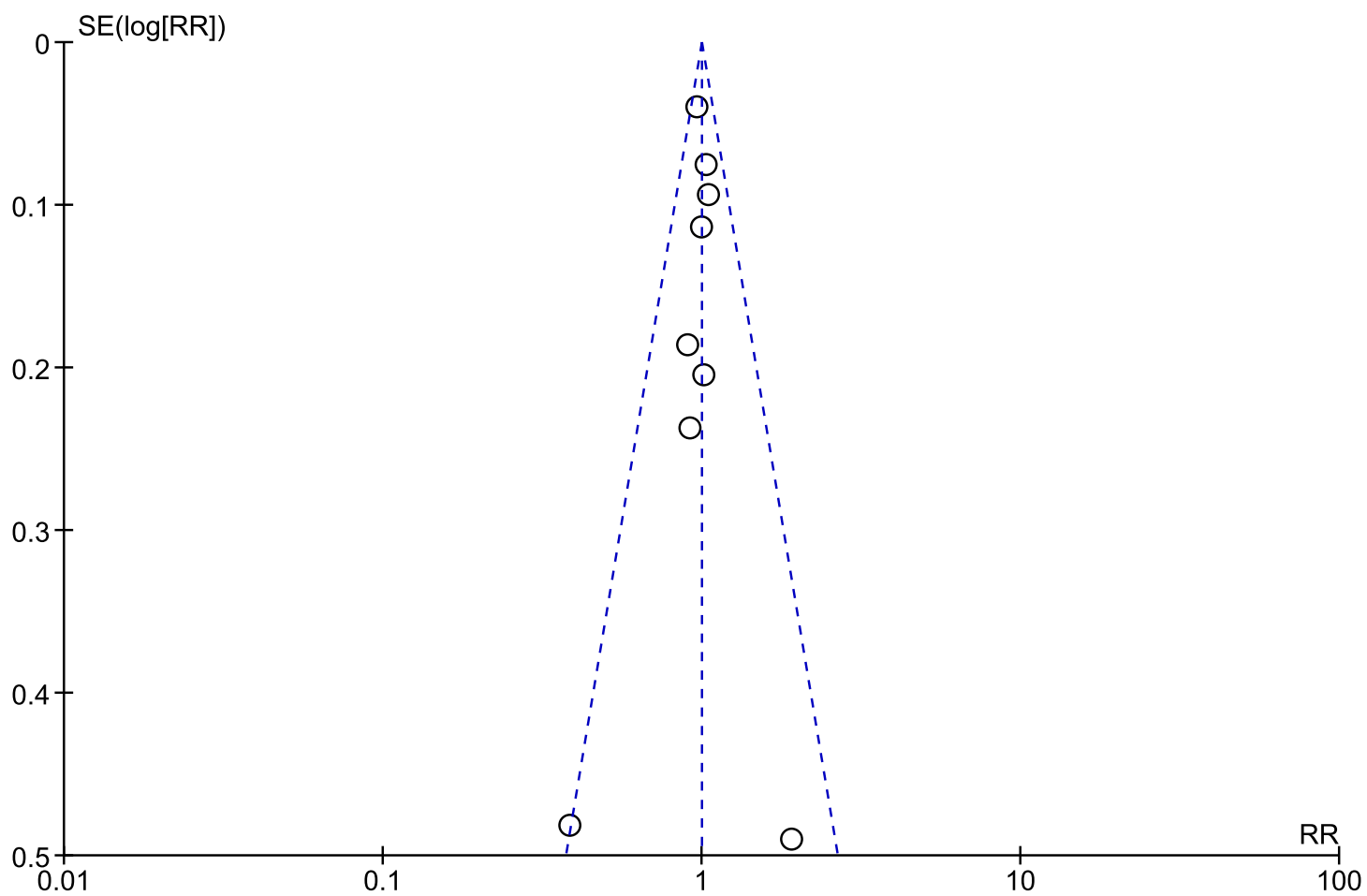

Figure S3. Hospital admission funnel plot.

Supplement: Supplementary file 3 — Additional file 3 Funnel plots (pdf). Figure S1. Medication appropriateness funnel plot (10studies). Figure S2. Medication appropriateness funnel plot (2 studies). Figure S3. Hospital admission funnel plot. Figure S4. Mortality funnel plot. Figure S5. Falls funnel plot. Figure S6. Quality of life (QoL) funnel plot. Figure S7. Behavioural and Psychological Symptoms of Dementia (BPSD) funnel plot. Figure S8. Adverse drug events (ADEs) funnel plot. Figure S9. Cognitive function funnel plot. [file 12877_2020_1634_MOESM3_ESM.zip › Figure S3. Hospital admission funnel plot R3.pdf]

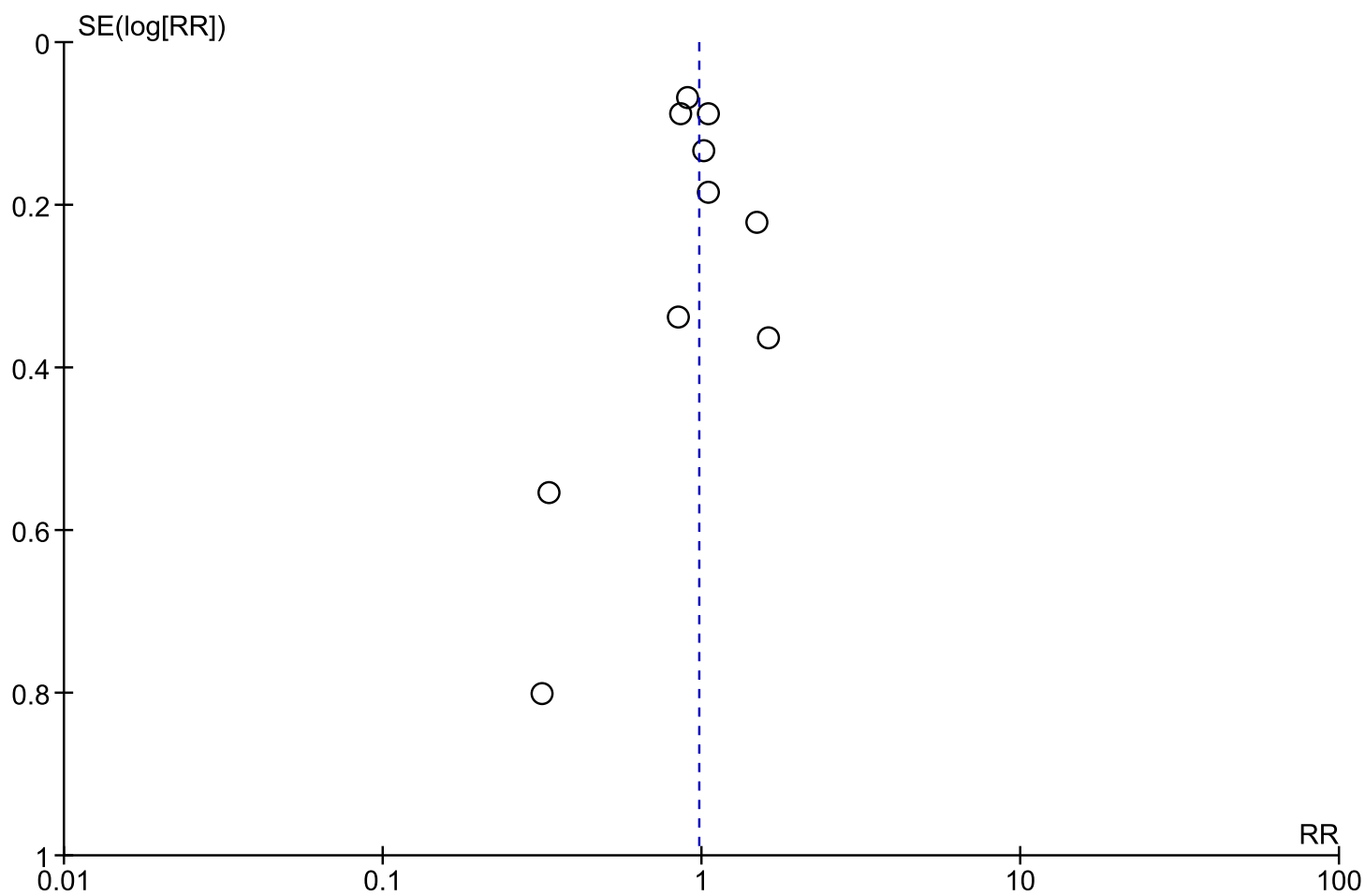

Figure S4. Mortality funnel plot.

Supplement: Supplementary file 3 — Additional file 3 Funnel plots (pdf). Figure S1. Medication appropriateness funnel plot (10studies). Figure S2. Medication appropriateness funnel plot (2 studies). Figure S3. Hospital admission funnel plot. Figure S4. Mortality funnel plot. Figure S5. Falls funnel plot. Figure S6. Quality of life (QoL) funnel plot. Figure S7. Behavioural and Psychological Symptoms of Dementia (BPSD) funnel plot. Figure S8. Adverse drug events (ADEs) funnel plot. Figure S9. Cognitive function funnel plot. [file 12877_2020_1634_MOESM3_ESM.zip › Figure S4. Mortality funnel plotR3.pdf]

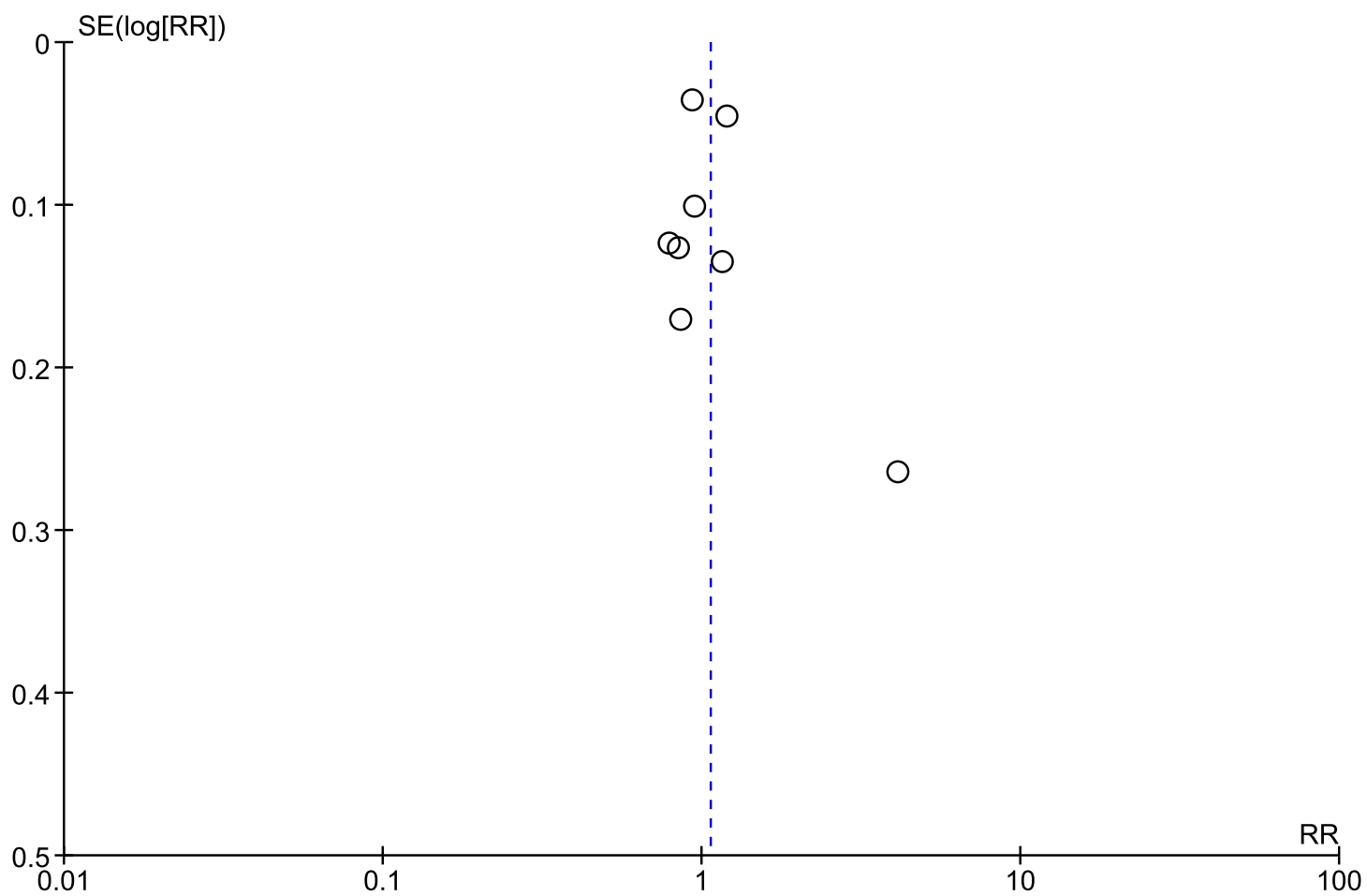

Figure S5. Falls funnel plot.

Supplement: Supplementary file 3 — Additional file 3 Funnel plots (pdf). Figure S1. Medication appropriateness funnel plot (10studies). Figure S2. Medication appropriateness funnel plot (2 studies). Figure S3. Hospital admission funnel plot. Figure S4. Mortality funnel plot. Figure S5. Falls funnel plot. Figure S6. Quality of life (QoL) funnel plot. Figure S7. Behavioural and Psychological Symptoms of Dementia (BPSD) funnel plot. Figure S8. Adverse drug events (ADEs) funnel plot. Figure S9. Cognitive function funnel plot. [file 12877_2020_1634_MOESM3_ESM.zip › Figure S5. Falls funnel plotR3.pdf]

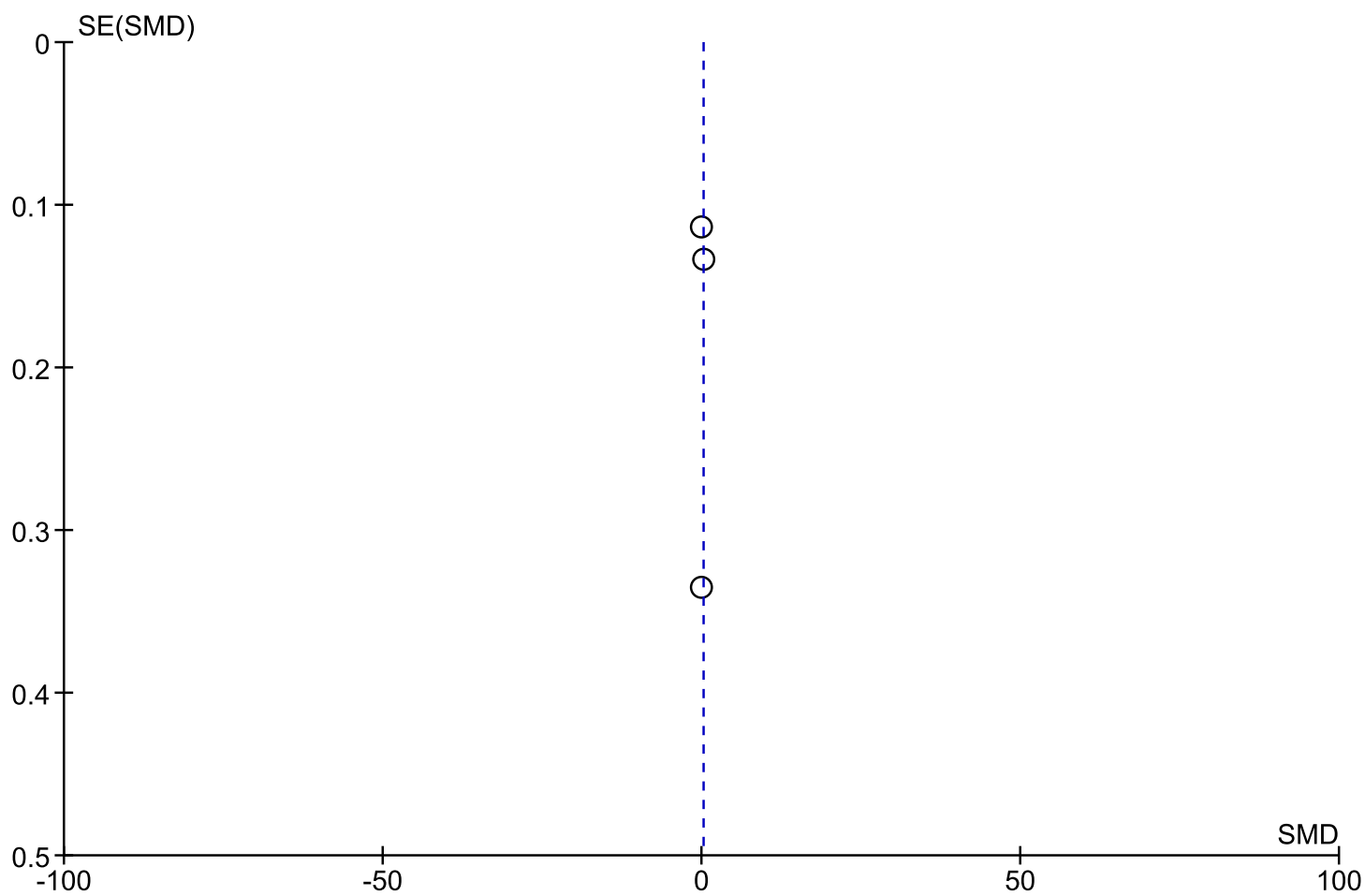

Figure S6. Quality of life (QoL) funnel plot.

Supplement: Supplementary file 3 — Additional file 3 Funnel plots (pdf). Figure S1. Medication appropriateness funnel plot (10studies). Figure S2. Medication appropriateness funnel plot (2 studies). Figure S3. Hospital admission funnel plot. Figure S4. Mortality funnel plot. Figure S5. Falls funnel plot. Figure S6. Quality of life (QoL) funnel plot. Figure S7. Behavioural and Psychological Symptoms of Dementia (BPSD) funnel plot. Figure S8. Adverse drug events (ADEs) funnel plot. Figure S9. Cognitive function funnel plot. [file 12877_2020_1634_MOESM3_ESM.zip › Figure S6. Quality of life (QoL) funnel plotR3.pdf]

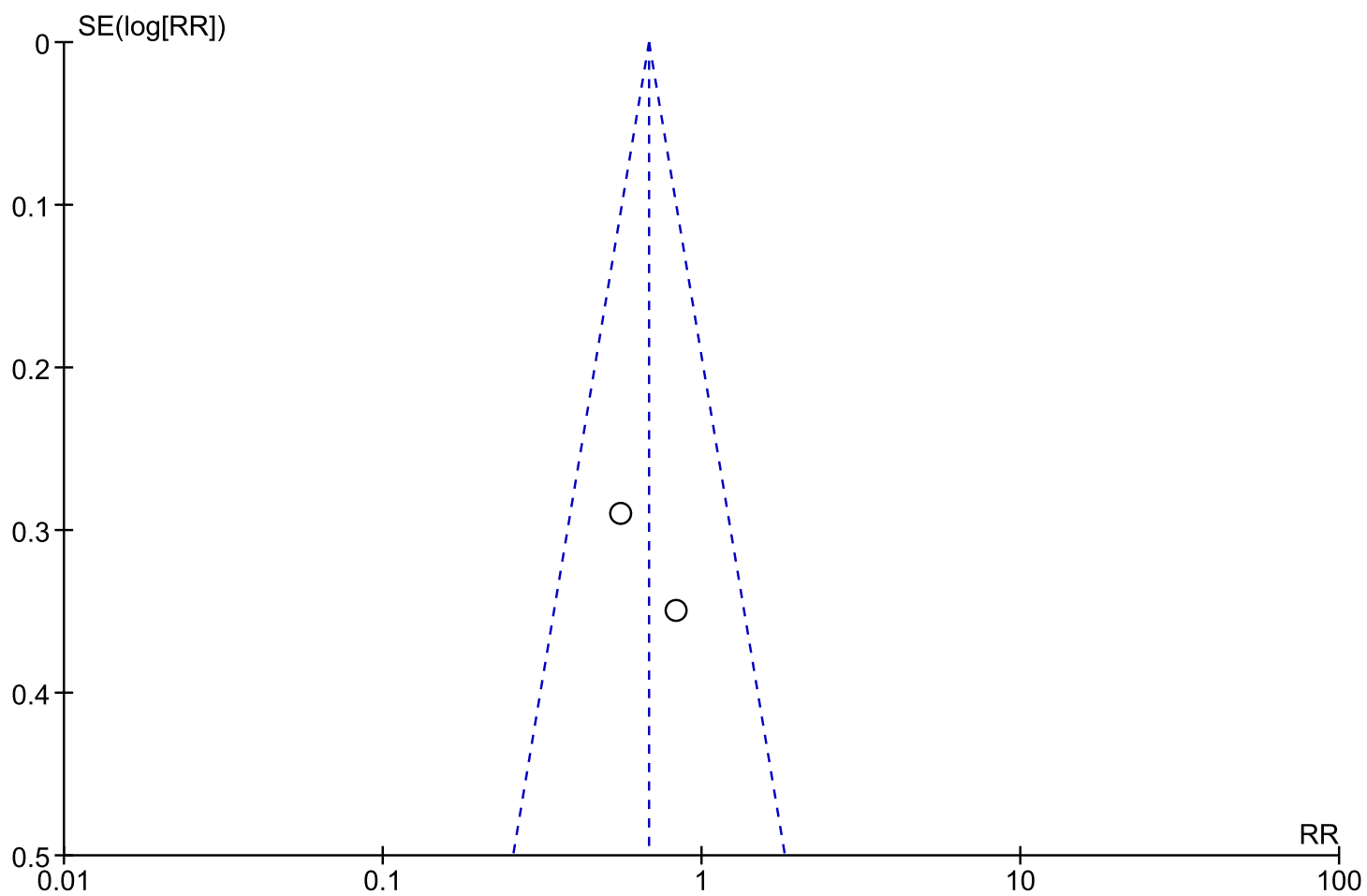

Figure S7. Behavioural and Psychological Symptoms of Dementia (BPSD) funnel plot.

Supplement: Supplementary file 3 — Additional file 3 Funnel plots (pdf). Figure S1. Medication appropriateness funnel plot (10studies). Figure S2. Medication appropriateness funnel plot (2 studies). Figure S3. Hospital admission funnel plot. Figure S4. Mortality funnel plot. Figure S5. Falls funnel plot. Figure S6. Quality of life (QoL) funnel plot. Figure S7. Behavioural and Psychological Symptoms of Dementia (BPSD) funnel plot. Figure S8. Adverse drug events (ADEs) funnel plot. Figure S9. Cognitive function funnel plot. [file 12877_2020_1634_MOESM3_ESM.zip › Figure S7. BPSD funnel plotR3.pdf]

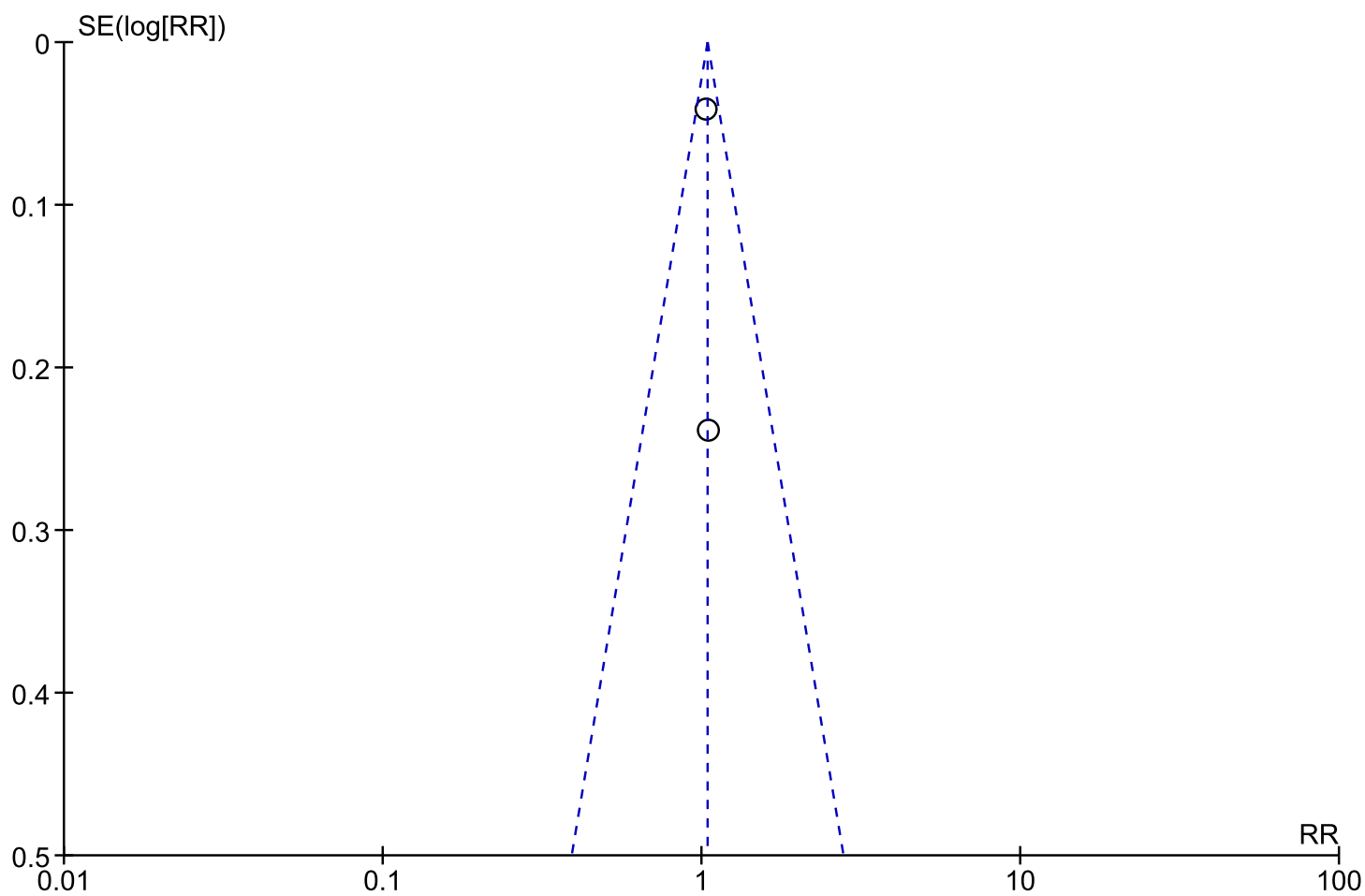

Figure S8. Adverse drug events (ADEs) funnel plot.

Supplement: Supplementary file 3 — Additional file 3 Funnel plots (pdf). Figure S1. Medication appropriateness funnel plot (10studies). Figure S2. Medication appropriateness funnel plot (2 studies). Figure S3. Hospital admission funnel plot. Figure S4. Mortality funnel plot. Figure S5. Falls funnel plot. Figure S6. Quality of life (QoL) funnel plot. Figure S7. Behavioural and Psychological Symptoms of Dementia (BPSD) funnel plot. Figure S8. Adverse drug events (ADEs) funnel plot. Figure S9. Cognitive function funnel plot. [file 12877_2020_1634_MOESM3_ESM.zip › Figure S8. Adverse drug events (ADEs) funnel plotR3.pdf]

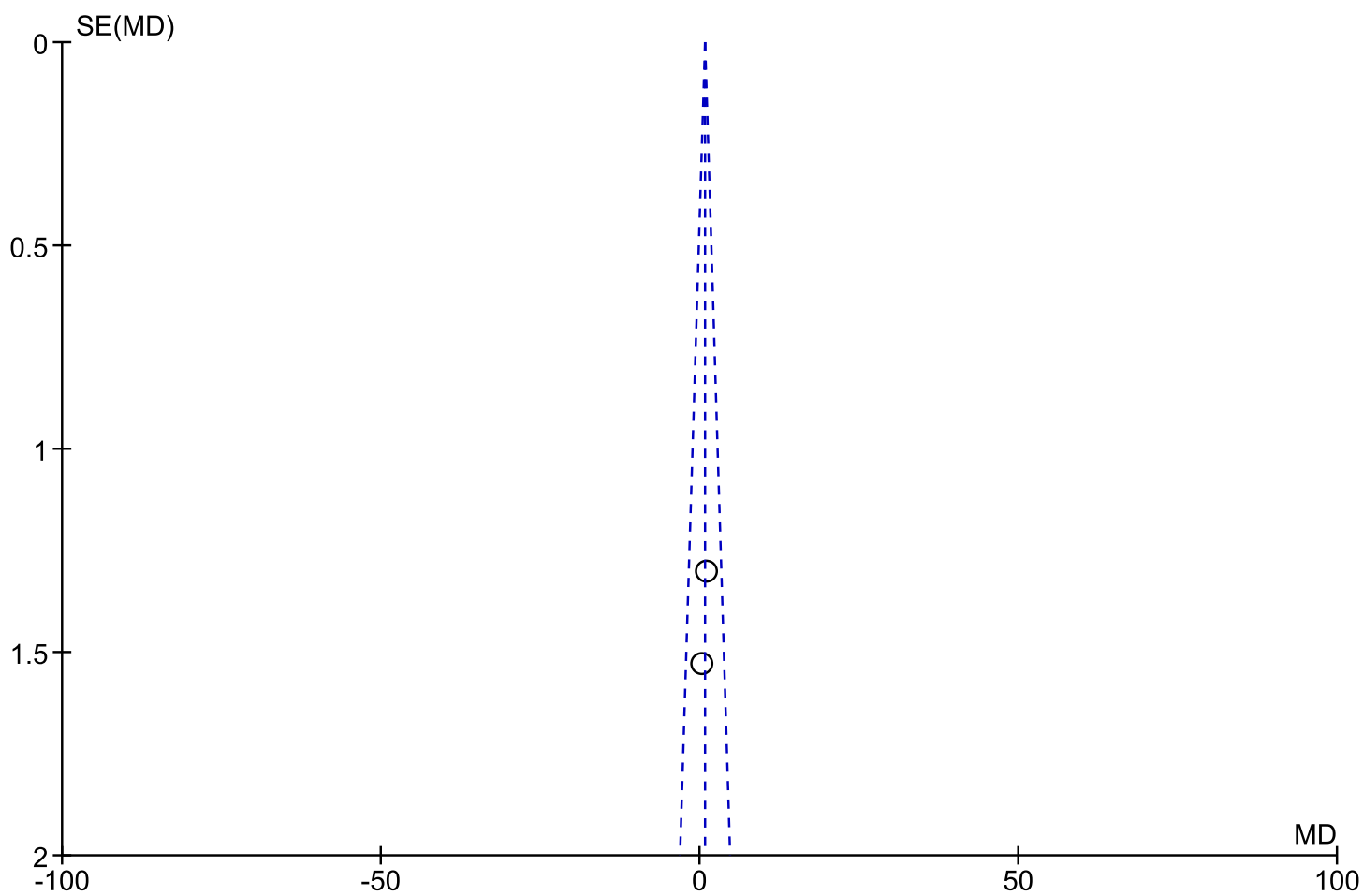

Figure S9. Cognitive function funnel plot.

Supplement: Supplementary file 3 — Additional file 3 Funnel plots (pdf). Figure S1. Medication appropriateness funnel plot (10studies). Figure S2. Medication appropriateness funnel plot (2 studies). Figure S3. Hospital admission funnel plot. Figure S4. Mortality funnel plot. Figure S5. Falls funnel plot. Figure S6. Quality of life (QoL) funnel plot. Figure S7. Behavioural and Psychological Symptoms of Dementia (BPSD) funnel plot. Figure S8. Adverse drug events (ADEs) funnel plot. Figure S9. Cognitive function funnel plot. [file 12877_2020_1634_MOESM3_ESM.zip › Figure S9. Cognitive function funnel plotR3.pdf]
